# Association between gut microbiota and NAFLD/NASH: a bidirectional two-sample Mendelian randomization study

**DOI:** 10.3389/fcimb.2023.1294826

**Published:** 2023-12-01

**Authors:** Qilong Zhai, Hongyu Wu, Siyuan Zheng, Tao Zhong, Changjie Du, Jiajun Yuan, Jialun Peng, Can Cai, Jinzheng Li

**Affiliations:** ^1^ Department of Hepatobiliary Surgery, The Second Affiliated Hospital of Chongqing Medical University, Chongqing, China; ^2^ Department of Gastroenterology, The Second Affiliated Hospital of Chongqing Medical University, Chongqing, China

**Keywords:** non-alcoholic fatty liver disease, non-alcoholic steatohepatitis, gut microbiota, Mendelian randomization, causality

## Abstract

**Background:**

Recent studies have suggested a relationship between gut microbiota and non-alcoholic fatty liver disease (NAFLD)/nonalcoholic steatohepatitis (NASH). However, the nature and direction of this potential causal relationship are still unclear. This study used two-sample Mendelian randomization (MR) to clarify the potential causal links.

**Methods:**

Summary-level Genome-Wide Association Studies (GWAS) statistical data for gut microbiota and NAFLD/NASH were obtained from MiBioGen and FinnGen respectively. The MR analyses were performed mainly using the inverse-variance weighted (IVW) method, with sensitivity analyses conducted to verify the robustness. Additionally, reverse MR analyses were performed to examine any potential reverse causal associations.

**Results:**

Our analysis, primarily based on the IVW method, strongly supports the existence of causal relationships between four microbial taxa and NAFLD, and four taxa with NASH. Specifically, associations were observed between Enterobacteriales (*P* =0.04), *Enterobacteriaceae* (*P* =0.04), *Lachnospiraceae UCG-004* (*P* =0.02), and *Prevotella9* (*P* =0.04) and increased risk of NAFLD. *Dorea* (*P* =0.03) and *Veillonella* (*P* =0.04) could increase the risks of NASH while *Oscillospira* (*P* =0.04) and *Ruminococcaceae UCG-013* (*P*=0.005) could decrease them. We also identified that NAFLD was found to potentially cause an increased abundance in *Holdemania* (*P* =0.007) and *Ruminococcus2* (*P* =0.002). However, we found no evidence of reverse causation in the microbial taxa associations with NASH.

**Conclusion:**

This study identified several specific gut microbiota that are causally related to NAFLD and NASH. Observations herein may provide promising theoretical groundwork for potential prevention and treatment strategies for NAFLD and its progression to NASH in future.

## 1 Introduction

Nonalcoholic fatty liver disease (NAFLD), currently the most prevalent liver disease worldwide, affects approximately 32.4% of adults, and its incidence is expected to continue rising, resulting in a significant clinical and economic burden (Riazi et al., 2022). Nonalcoholic steatohepatitis (NASH), which represents a progressive stage of NAFLD, may lead to liver cirrhosis, and is directly linked to an increased risk of liver cancer (Younossi et al., 2023). Almost 16.02% of NAFLD patients eventually develop NASH (Younossi et al., 2023), so it’s crucial to identify the risk factors associated with NAFLD/NASH onset and progression.

NAFLD/NASH results from an interplay of multiple factors, including a genetic predisposition, obesity, insulin resistance, and an inflammatory cascade reaction (Tilg and Moschen, 2010). In the past few years, scientists have found that dysbiosis in the gut microbiota are intimately connected to NAFLD pathogenesis and development (Fang et al., 2022). Gut dysbiosis can increase endogenous ethanol, short-chain fatty acids (SCFAs), and fasting-induced adipose factor (FIAF) levels while reducing choline levels (Campo et al., 2019). When the intestinal barrier function is compromised, these factors can interfere with the liver through the gut-liver axis, potentially leading to NAFLD, including liver steatosis, NASH, and ultimately, cirrhosis. Additionally, dysbiosis can trigger endotoxemia, activate Toll-like receptors 9 (TLR9) and 4 (TLR4) in Kupffer and stellate cells, and stimulate TNF-α production, further contributing to NAFLD development (Campo et al., 2019).

Currently, research indicates that the composition and abundance of gut microbiota change significantly at the phylum, class, family, and genus levels among NAFLD/NASH patients, compared to healthy individuals (Aron-Wisnewsky et al., 2020). In addition, the abundance of gut microbiota varies among subjects with varying degrees of liver fibrosis (Gomez-Perez et al., 2023). However, the current studies are designed as cross-sectional and observational studies with limited sample size and confounding factors. The changes in gut microbiota reported in different studies have various differences at the level of phylum, genus, and family, which makes the study results difficult to reproduce. Furthermore, patients with NAFLD often have metabolism-related complications such as obesity and type 2 diabetes, which can bias the results (Moszak et al., 2020). While the association of gut microbiota with NAFLD/NASH is evident, it is also possible that NAFLD/NASH influence gut microbiota due to the intricate interplay between the gut and liver. As such, the specific cause-effect relationship between NAFLD/NASH and gut microbiota requires further exploration.

Mendelian randomization (MR), which is a tool leveraging genetic variants linked to modifiable exposures to investigate their potential causal relationship with outcomes, offers a possible solution to the issues of confounding and reverse causality. It has already been applied in studying the relationship between gut microbiota and various diseases (Ponziani et al., 2019; Li et al., 2023; Zeng et al., 2023). Our primary aim is to investigate the causal link between gut microbiota and NAFLD/NASH, thereby building a solid theoretical foundation for understanding the NAFLD/NASH pathogenesis, using the bidirectional two-sample MR approach.

## 2 Methods

### 2.1 Data sources

#### 2.1.1 Exposure GWAS: gut microbiota

The human gut microbiome genome-wide association study (GWAS) dataset utilized in this research was sourced from the largest meta-analysis of microbiota composition to date, conducted by the MiBioGen consortium (Kurilshikov et al., 2021). The MiBioGen consortium was established to investigate host-genetic-microbiome associations, and represents the largest multi-ethnic genome-wide analysis of its kind. The cohort comprised 18,340 individuals from 24 distinct cohorts, with 13,266 participants of European ancestry (Kurilshikov et al., 2021). Microbiome composition was analyzed and classified for the V4, V3-V4, and V1-V2 variable regions of the 16S rRNA gene. Sequencing profiles and genotyping data were obtained through mapping and analysis of microbiota quantitative trait loci (mbQTL), resulting in the identification of 122,110 genetic loci associated with bacterial taxon abundance levels in the gut microbiota. The study encompassed 211 bacterial taxa (131 genera, 35 families, 20 orders, 16 classes, and 9 phyla), with genus serving as the lowest taxonomic level.

#### 2.1.2 Outcome GWAS: NAFLD and NASH

The GWAS summary statistics used in this research for NAFLD and NASH were obtained from the FinnGen Consortium R9 release data, which were made publicly available in May of 2023. The NAFLD GWAS summary statistics were derived from a cohort of 377,277 Finnish adults, comprising 2,275 cases and 375,002 controls. GWAS summary statistics for NASH included 157 cases and 377,120 controls. Further information regarding the cohorts, genotypes, endpoint definitions, and association tests used in the FinnGen consortium is available through the FinnGen webpage.

### 2.2 Instrumental variable selection

To ensure the precision of our findings, we performed a screening of the extracted gut microbiota data. Firstly, we established a gene locus significance threshold for gut microbiota of *P <*10^-5^, as reported by Sanna et al. (2019) (Sanna et al., 2019). Secondly, we employed data from the 1000 Genomes project European samples as the reference panel for calculating linkage disequilibrium (LD) between single nucleotide polymorphisms (SNPs), and subsequently removed SNPs with LD (r^2^<0.01, clumping window size=10,000kb). Additionally, we excluded SNPs with minor allele frequencies (MAF) <0.01 and palindromic SNPs. Finally, we employed the F-statistic to evaluate the strength of the instrumental variables (IVs). This metric is derived using a formula [F = R^2^×(N-k-1)/k×(1-R^2^)] that accounts for a variety of factors, including total variance (R^2^), sample size (N), and the number of IVs included in the analysis (k) (Noyce et al., 2017). Weak genetic instruments were defined as those with F-statistics <10 and were thus excluded from our analysis (Burgess and Thompson, 2011).

### 2.3 Statistical analysis

Mendelian randomization analysis, a cutting-edge methodology that has been gaining traction in recent years, owing to its unique ability to circumvent the limitations of traditional research methods. At its core, MR analysis leverages exposure-related genetic variation as IVs, in order to investigate the causal effects of modifiable exposures on outcomes (Richmond and Davey Smith, 2022). Thanks to the fortuitous nature of genetic variants, which are randomly assigned at the time of conception, MR analysis is able to sidestep the thorny issues of confounding factors and reverse causality, which have plagued other research methods in the past (Davey Smith and Hemani, 2014). By exploiting this inherent randomness, MR analysis is able to offer a more robust and reliable means of establishing causality.

**Figure 1:**
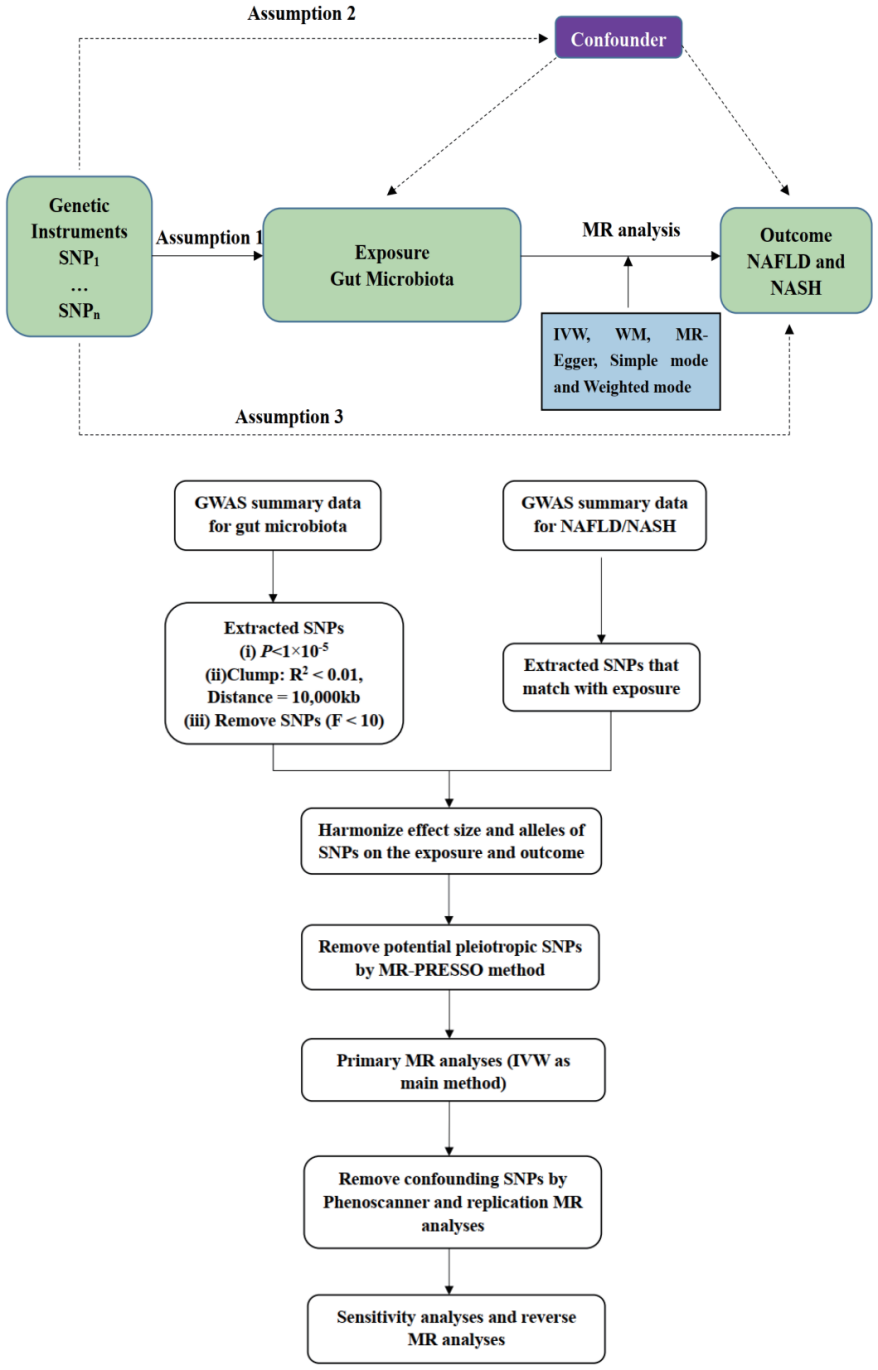
Three assumptions and flowchart of the MR analyses.

To ensure the validity of our results, we had to adhere to a rigorous set of assumptions (shown in **Figure 1**). These assumptions included a strong association between our IVs and the exposure factors under investigation, as well as a complete lack of association between these IVs and the outcomes we were studying. Moreover, we had to be certain that our IVs were not influenced by any confounding factors that might distort the results of our study.

In this study, we employed five different MR methods, including inverse-variance weighted (IVW), weighted median (WM), MR‐Egger, simple mode, and weighted mode, to assess the causal effects of gut microbiota on NAFLD and NASH. These MR methods have varying assumptions regarding horizontal pleiotropy. Various statistical methods were used in this study. As the IVW method is used to estimate causality through a meta-analysis of Wald values, the IVW method was used as the main method (Hartwig et al., 2017). In order to further bolster the credibility of our results, we also employed two additional methods – the WM method and the MR-Egger method – and compared the results obtained through each of these methods against the results obtained using the IVW method.

The WM method was used to exclude invalid instruments, with a maximum allowable proportion of 50% (Bowden et al., 2016). In contrast, the MR-Egger method allowed all instruments to be invalidated and was used to evaluate potential directional pleiotropy by examining the intercept term (Bowden et al., 2015). The simple mode and weighted mode methods were employed as complementary approaches to the primary three methods. A significance level of *P <*0.05 was employed, with MR estimates presented as odds ratios (OR) with 95% confidence intervals (CI). Overall, the five different MR methods utilized in this study were chosen based on their underlying assumptions regarding horizontal pleiotropy, and were employed to investigate the causal relationships between gut microbiota and disease.

Furthermore, we conducted an evaluation of whether IVs were correlated with risk factors for NAFLD and NASH using phenoscanner (http://www.phenoscanner.medschl.cam.ac.uk/). To avoid potential confounding factors from impacting the ultimate causality, we eliminated SNPs linked to NAFLD or NASH risk factors and subsequently re-conducted the MR analysis.

To provide additional clarity regarding the genuine causal association between gut microbiota and NAFLD and NASH, we conducted a reverse MR analysis. Specifically, we employed NAFLD and NASH-related GWAS data as exposures, with known causative genera serving as outcomes. SNPs linked to NAFLD and NASH at the genome-wide significance threshold of *P <*5.0×10^−6^ were regarded as potential IVs, with the remaining statistical parameters being consistent with those employed in the forward MR analysis. The reverse MR analysis was utilized to investigate whether NAFLD and NASH exerted any causal impact on the identified critical bacterial genera. A flowchart of the MR study is presented in **Figure 1**.

### 2.4 Sensitivity analyses

To evaluate the significance of our findings, we performed a variety of sensitivity analyses. Cochran’s Q test was applied to assess heterogeneity among SNPs associated with each microbial taxon. Leave-one-out analysis was conducted to evaluate the influence of individual SNPs on the overall estimate, thereby identifying any anomalous instrumental variables that significantly impacted the estimation of causal effects (Chen et al., 2021). In addition, we employed MR-Egger regression and MR-PRESSO to evaluate horizontal pleiotropy (Ong and MacGregor, 2019). MR-Egger regression enabled us to assess whether the instrumental variables had a pleiotropic effect on the results. If the MR-Egger intercept had a p-value >0.05, each SNP met the Mendelian hypothesis, and the results obtained using IVW were deemed reliable. Conversely, if the intercept had a p-value <0.05, the instrumental variables indicated potential directional pleiotropy. MR-PRESSO had greater precision and statistical efficacy than MR-Egger, and identified and corrected for the effects of heterogeneous outliers in the instrument. It eliminated the impact of horizontal pleiotropy on the final results by removing outliers and obtaining the most realistic causality.

All of the above statistical analyses and data visualizations were carried out using the R packages “TwoSample MR” and “MRPRESSO” and are available in R software (version 4.2.0) (Hemani et al., 2018).

## 3 Results

### 3.1 Selection of instrument variables

The baseline information for NAFLD, NASH, and microbiome cohorts was shown in **Supplementary Table 1**. After implementing a series of quality control procedures, a total of 14,587 SNPs were utilized as instrumental variables for 211 clusters. Initial MR analysis revealed that NAFLD was correlated with 7 distinct microbial taxa, involving 84 SNPs. All SNPs associated with gut microbiota had F-statistics exceeding 10, suggesting that the results were unlikely to be impacted by weak instrumental bias. Furthermore, we identified 18 SNPs linked to NAFLD risk factors through phenoscanner, and subsequently conducted MR analysis again following the exclusion of these confounding instrumental variables. Similarly, we obtained a causal relationship between 8 colonies and NASH by preliminary MR analysis, involving a total of 96 SNPs. We also performed MR analysis again after excluding 20 SNPs associated with NASH confounders.

### 3.2 Data analysis

#### 3.2.1 Gut Microbiota and NAFLD

**Figure 2:**
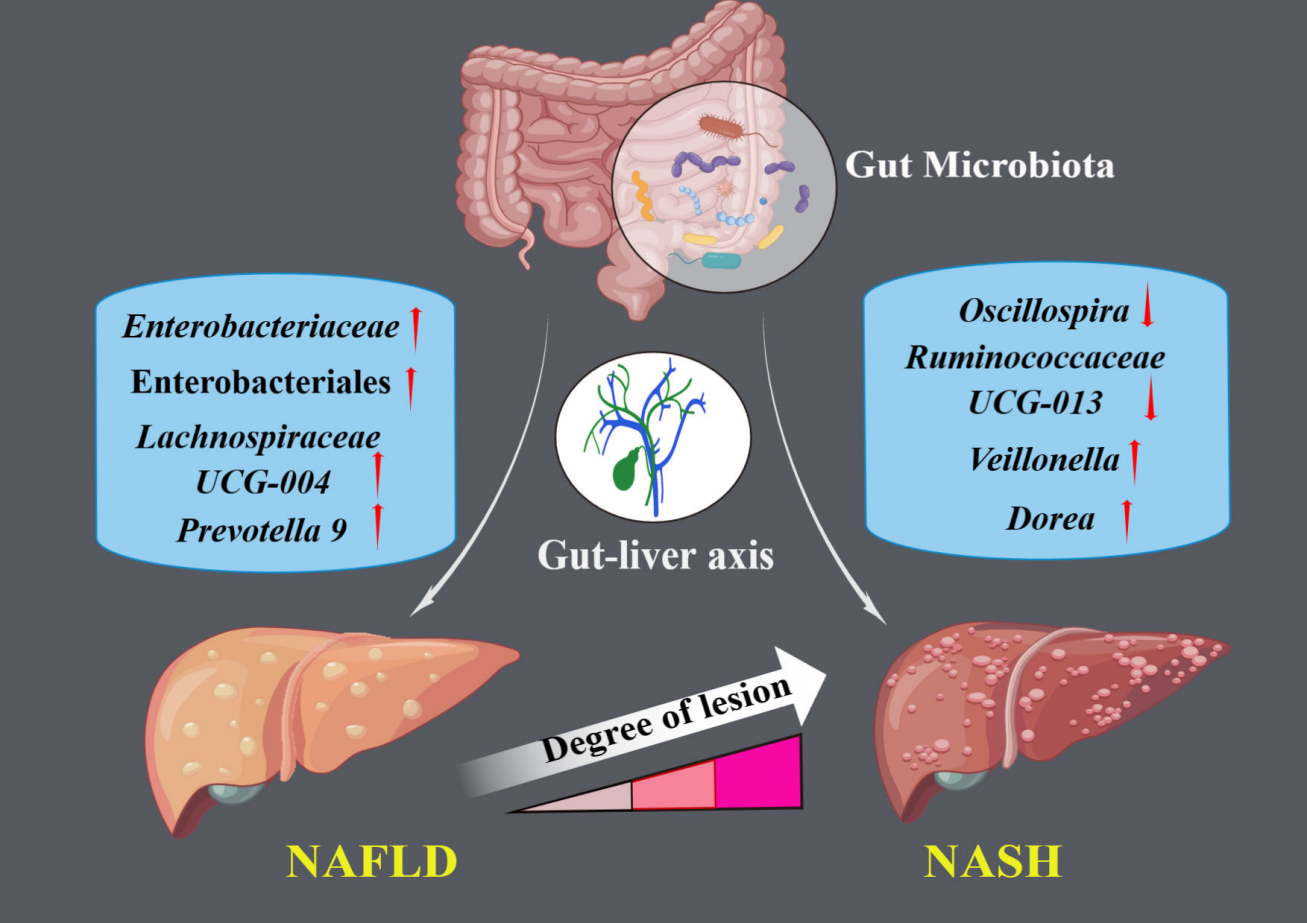
The illustration represents significant causal gut microbial taxa associated with NAFLD and NASH.

**Figure 3:**
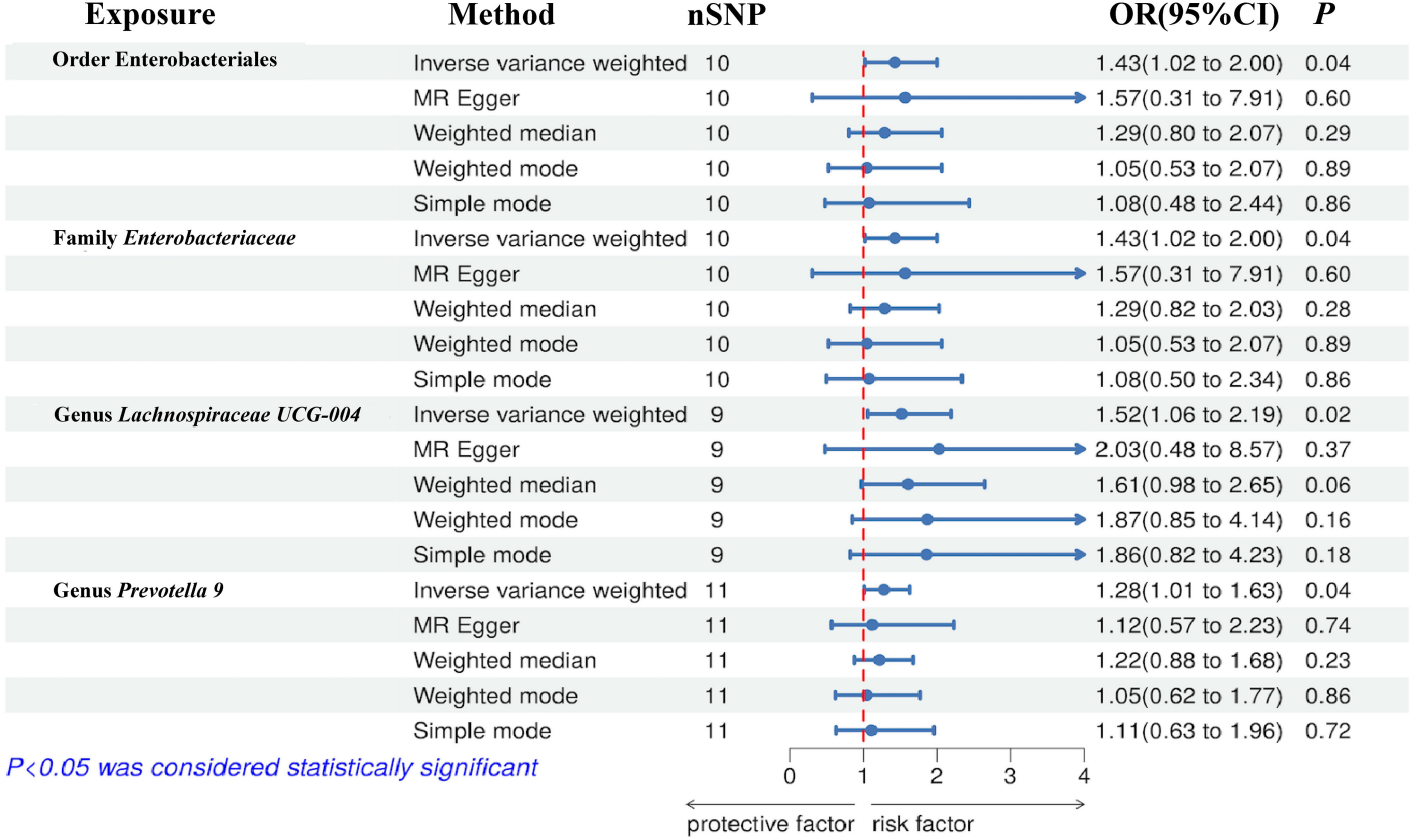
Forest plot of the associations between genetically determined gut microbial taxa with the risks of NAFLD.

**Figure 4:**
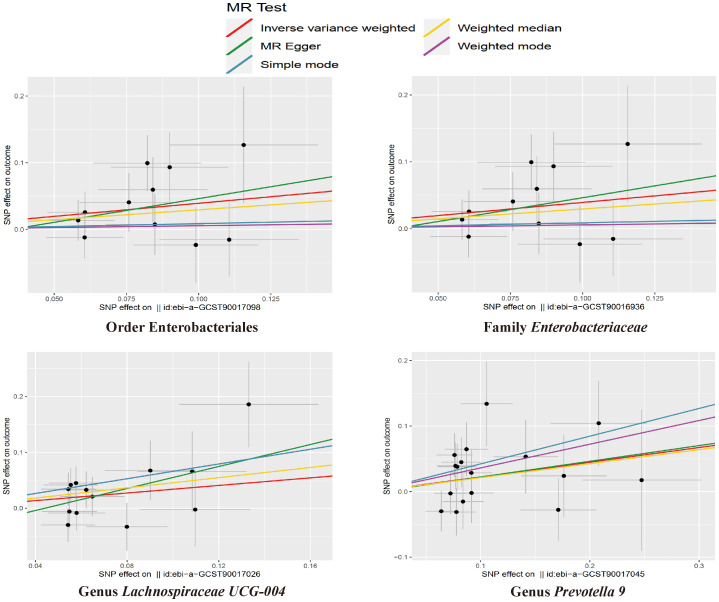
The scatter plots for the causal association between gut microbial taxa and NAFLD.

**Figure 5:**
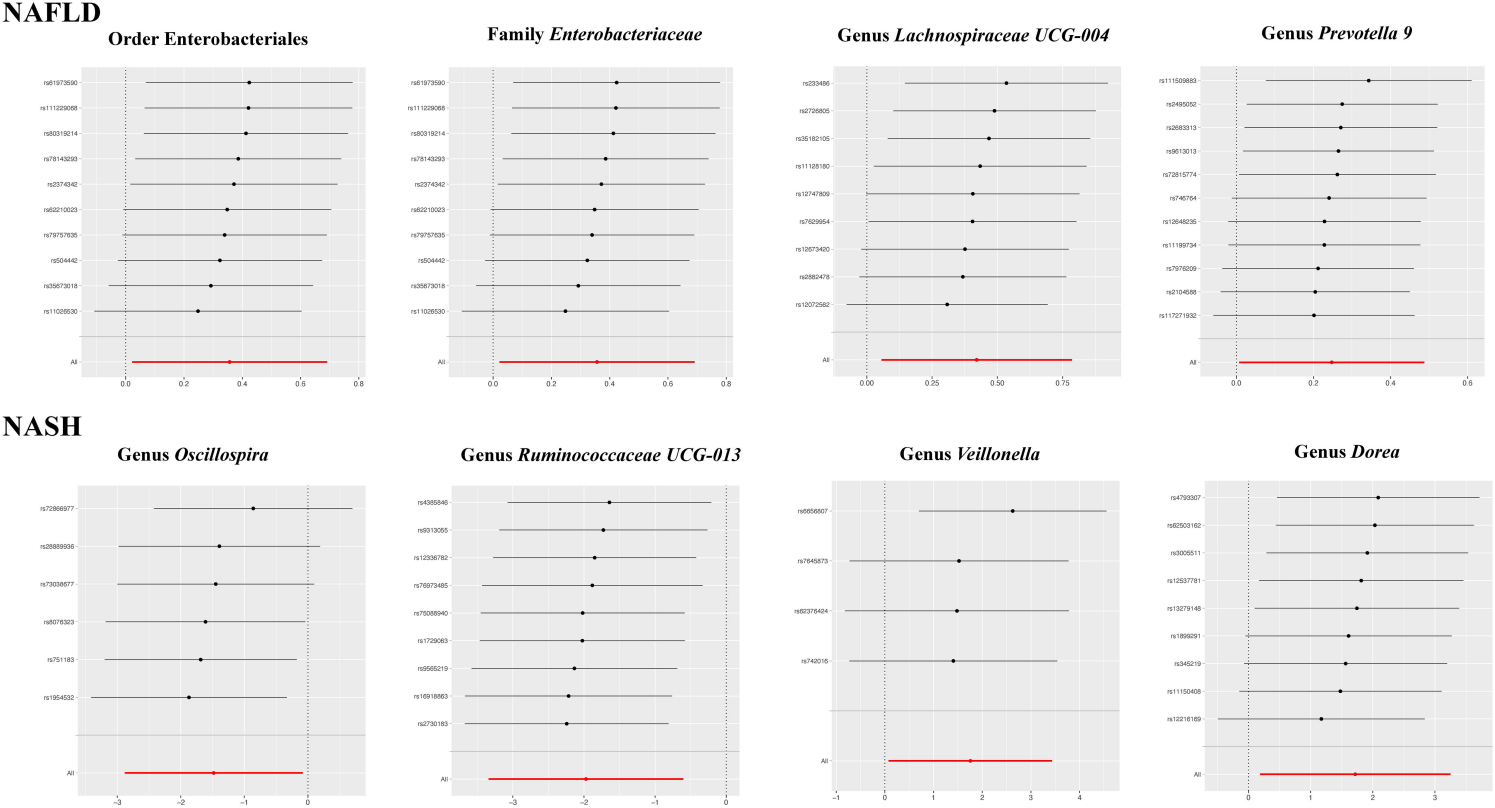
The leave-one-out plots for the causal association between gut microbial taxa and NAFLD/NASH.

The results of the IVW test indicated that the genetically predicted abundance of seven microbial taxa, specifically Gammaproteobacteria, *Enterobacteriaceae*, *Lachnospiraceae UCG-004*, *Prevotella7*, *Prevotella9*, Desulfovibrionales, and Enterobacteriales, exhibited significant associations with increased or decreased risk of NAFLD in terms of relative abundance. Initially, our preliminary MR analysis revealed an inverse relation between the risk of NAFLD and the relative abundance of Gammaproteobacteria as predicted genetically (OR = 0.62, 95% CI: 0.41-0.93, *P* = 0.02). This protective causality became insignificant after we removed a single SNP (rs75101789) using phenoscanner (OR = 0.68, 95% CI: 0.44-1.04, *P* = 0.07). Genetically predicted *Enterobacteriaceae* abundance was positively linked to an increased risk of NAFLD (OR = 1.48, 95% CI: 1.07-2.05, *P* = 0.02), with this causal relationship remaining significant even after excluding SNPs associated with NAFLD risk factors (OR = 1.43, 95% CI: 1.02-2.00, *P* = 0.04). Higher gene-predicted *Lachnospiraceae UCG-004* abundance was also associated with elevated NAFLD risk (OR = 1.40, 95% CI: 1.04-1.90, *P* = 0.03), with consistent findings observed in the repeated MR analysis that excluded confounding SNPs (OR = 1.52, 95% CI: 1.06-2.19, *P* = 0.02). The original MR analysis suggested that people with higher abundance of *Prevotella7* have lower NAFLD risks (OR = 0.83, 95% CI: 0.71-0.97, *P* = 0.02), but this causal relationship became less significant in the repeated MR analysis after excluding confounding SNPs (OR = 0.86, 95% CI: 0.71-1.03, *P* = 0.10). However, higher gene-predicted *Prevotella9* abundance was significantly associated with increased NAFLD risk (OR = 1.25, 95% CI: 1.03-1.53, *P* = 0.03), with this causal relationship remaining significant even after excluding SNPs linked to NAFLD risk factors (OR = 1.28, 95% CI: 1.01-1.63, *P* = 0.04). The increased abundance of Desulfovibrionales may reduce the risk of NAFLD (OR = 0.71, 95% CI: 0.52-0.98, *P* = 0.04) following MR analysis, with consistent findings observed in both IVW and WM tests. However, this protective causality did not hold after excluding confounding SNPs (OR = 0.72, 95% CI: 0.50-1.02, *P* = 0.07). Finally, genetically predicted Enterobacteriales abundance was associated with an elevated risk of NAFLD (OR = 1.48, 95% CI: 1.07-2.05, *P* = 0.02), with this causal relationship remaining significant even after excluding SNPs associated with NAFLD risk factors (OR = 1.43, 95% CI: 1.02-2.00, *P* = 0.04). The gut microbiota obtained after screening for causal association with NAFLD are shown in **Figure 2**. MR estimate for the association between gut microbiota and NAFLD was shown in **Table 1** and **Figure 3**. The scatter plots showed the causal relationship between gut microbiota and NAFLD in the five MR Methods (**Figure 4**). Sensitivity analyses, such as Cochran’s Q test, MR-Egger, and MR-PRESSO (shown in **Supplementary Table 2**), did not provide evidence of pleiotropy (P > 0.05). No abnormal SNPs were identified in Leave-one-out analysis, indicating that the identified causal relationships were not driven by a single SNP (**Figure 5**).

**Table 1:** MR estimate for the association between gut microbiota and NAFLD.

Bacterial taxa (Exposure)	MR method	SNP(N)	F-statistic	OR	95%CI	*P* value
Enterobacteriales	Inverse variance weighted	10	30.7	1.43	1.02-2.00	0.04
	MR Egger	10		1.57	0.31-7.91	0.60
	Weighted median	10		1.29	0.80-2.07	0.29
	Weighted mode	10		1.05	0.53-2.07	0.89
	Simple mode	10		1.08	0.48-2.44	0.86
*Enterobacteriaceae*	Inverse variance weighted	10	30.2	1.43	1.02-2.00	0.04
	MR Egger	10		1.57	0.31-7.91	0.60
	Weighted median	10		1.29	0.82-2.03	0.28
	Weighted mode	10		1.05	0.53-2.07	0.89
	Simple mode	10		1.08	0.50-2.34	0.86
*Lachnospiraceae UCG-004*	Inverse variance weighted	9	27.9	1.52	1.06-2.19	0.02
	MR Egger	9		2.03	0.48-8.57	0.37
	Weighted median	9		1.61	0.98-2.65	0.06
	Weighted mode	9		1.87	0.85-4.14	0.16
	Simple mode	9		1.86	0.82-4.23	0.18
*Prevotella9*	Inverse variance weighted	11	48.1	1.28	1.01-1.63	0.04
	MR Egger	11		1.12	0.57-2.23	0.74
	Weighted median	11		1.22	0.88-1.68	0.23
	Weighted mode	11		1.05	0.62-1.77	0.86
	Simple mode	11		1.11	0.63-1.96	0.72

MR, Mendelian randomization; SNP, Single nucleotide polymorphisms; N, Numbers; CI, Confidence interval.

**Figure 6:**
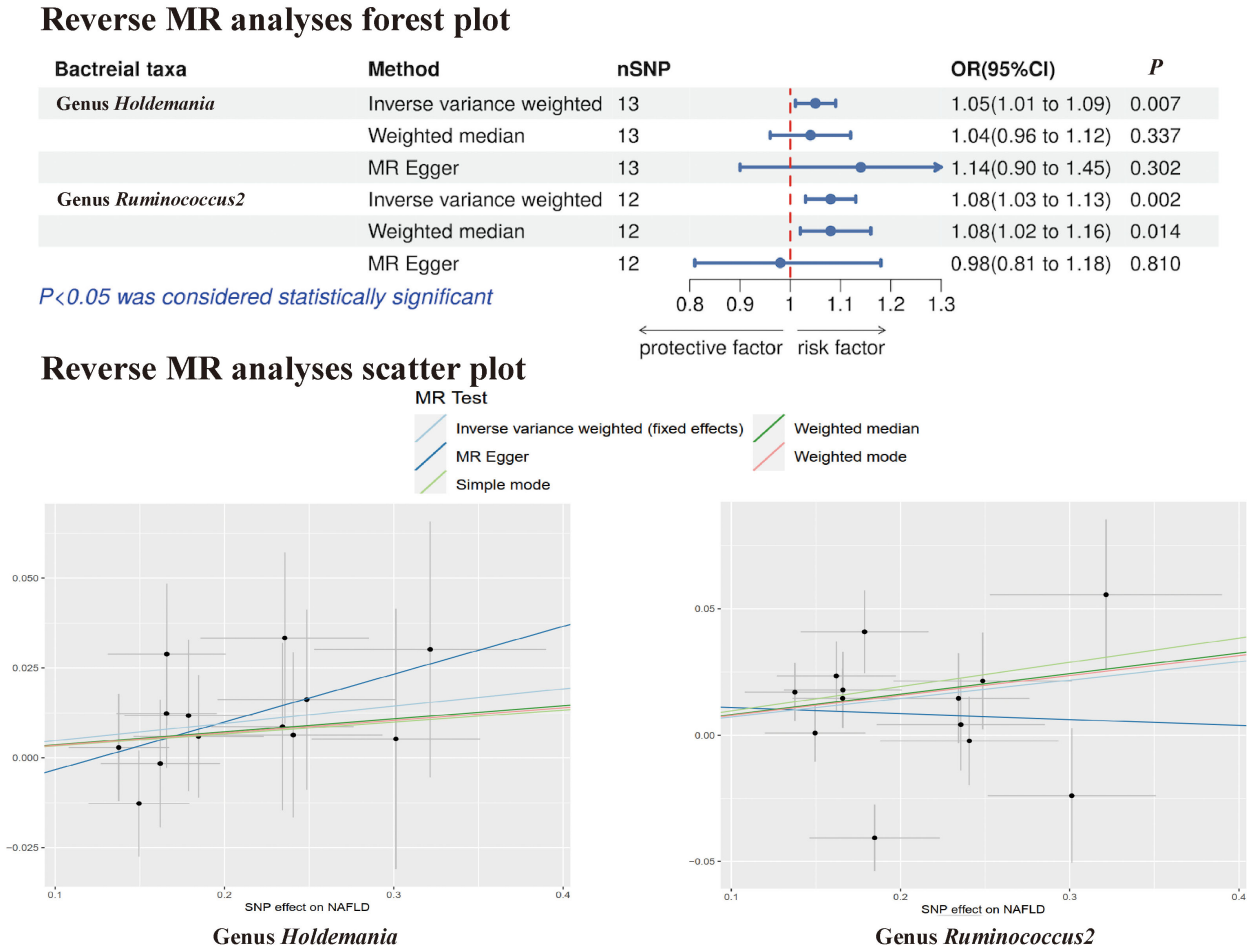
The Forest plot and Scatter plot of Reverse MR.

In the reverse MR analysis, no evidence of a causal effect of NAFLD on the aforementioned microbial taxa was observed. This suggests that the results of our MR analyses are not affected by reverse causation. Interestingly, in the reverse MR analysis, we found that NAFLD appeared to influence the abundance of 2 gut microbial taxa (genus *Holdemania*, genus *Ruminococcus2*). The reverse MR analysis showed that NAFLD could increase the abundance of the genus *Holdemania* (OR = 1.05, 95% CI: 1.01-1.09, *P* = 0.007) and genus *Ruminococcus2* (OR = 1.08, 95% CI: 1.03-1.13, *P* = 0.002), and these results were shown in **Supplementary Tables 3**, **4** and **Figure 6**.

#### 3.2.2 Gut microbiota and NASH

The preliminary MR analyses revealed a causal relationship between the relative abundance of eight genetically predicted gut microbial taxa (*Dorea*, *Oscillospira*, *Ruminococcaceae UCG-013*, *Ruminococcaceae UCG-014*, *Ruminococcus1*, unknown genus (id.2041), *Veillonella*, Rhodospirillales) and NASH. Specifically, increased relative abundance of *Dorea* was found to be associated with an increased risk of NASH, as determined by the primary outcome of the IVW method (OR = 4.08, 95% CI: 1.12-14.85, *P* = 0.03), with similar results obtained from other methods such as WM. This causality remained significant even after excluding SNPs associated with risk factors (OR = 5.57, 95% CI: 1.20-25.76, *P* = 0.03). Conversely, raw MR analysis suggested that the increased abundance of *Oscillospira* may reduce the risk of NASH (OR = 0.21, 95% CI: 0.07-0.65, *P* = 0.006), a relationship that remained significant after excluding SNPs associated with risk factors (OR = 0.23, 95% CI: 0.06-0.92, *P* = 0.04). Our study also showed that the increased abundance of *Ruminococcaceae UCG-013* (OR = 0.21, 95% CI: 0.07-0.65, *P* = 0.007) and *Ruminococcus1* (OR = 0.31, 95% CI: 0.11-0.91, *P* = 0.03) can reduce the risk of NASH, whereas *Ruminococcaceae UCG-014* (OR = 2.66, 95% CI: 1.06-6.67, *P* = 0.04) was a risk factor for NASH. However, the genetically predicted *Ruminococcaceae UCG014* (OR = 2.54, 95% CI: 0.97-6.64, *P* = 0.06) and *Ruminococcus1* (OR = 0.29, 95% CI: 0.08-1.02, *P* = 0.053) showed an insignificant relationship with NASH after excluding confounding factors while *Ruminococcaceae UCG-013* still showed a significant relationship with NASH (OR = 0.139, 95% CI: 0.04-0.55, *P* = 0.005). Additionally, an unknown genus (id.2041) appeared to be protective against NASH, but subsequent MR analyses did not yield significant results (OR = 0.47, 95% CI: 0.18-1.22, *P* = 0.12). Finally, we found that elevated relative abundance of *Veillonella* (OR = 5.79, 95% CI: 1.08-31.10, *P* = 0.004) and Rhodospirillales (OR = 2.82, 95% CI: 1.26-6.32, *P* = 0.01) was associated with an increased risk of NASH. However, after excluding confounders, this causal relationship between *Veillonella* and NASH remained significant (OR = 5.79, 95% CI: 1.08-31.10, *P* = 0.04), but this causal relationship betewen Rhodospirillales and NASH was not statistically significant (OR = 2.12, 95% CI: 0.90-4.98, *P* = 0.08). The gut microbiota obtained after screening for causal association with NASH are shown in **Figure 2**.

**Figure 7:**
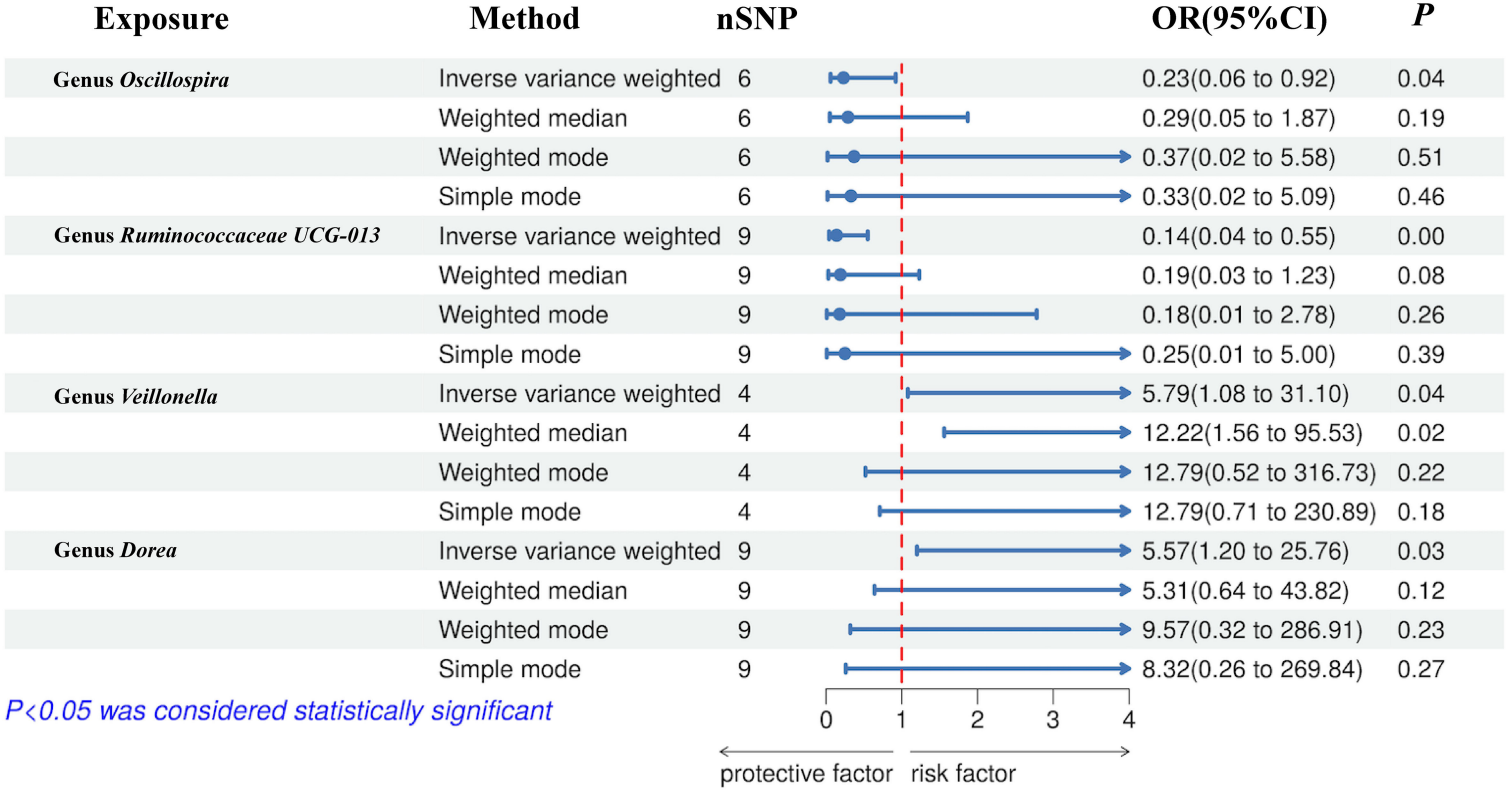
Forest plot of the associations between genetically determined gut microbial genera with the risks of NASH.

**Figure 8:**
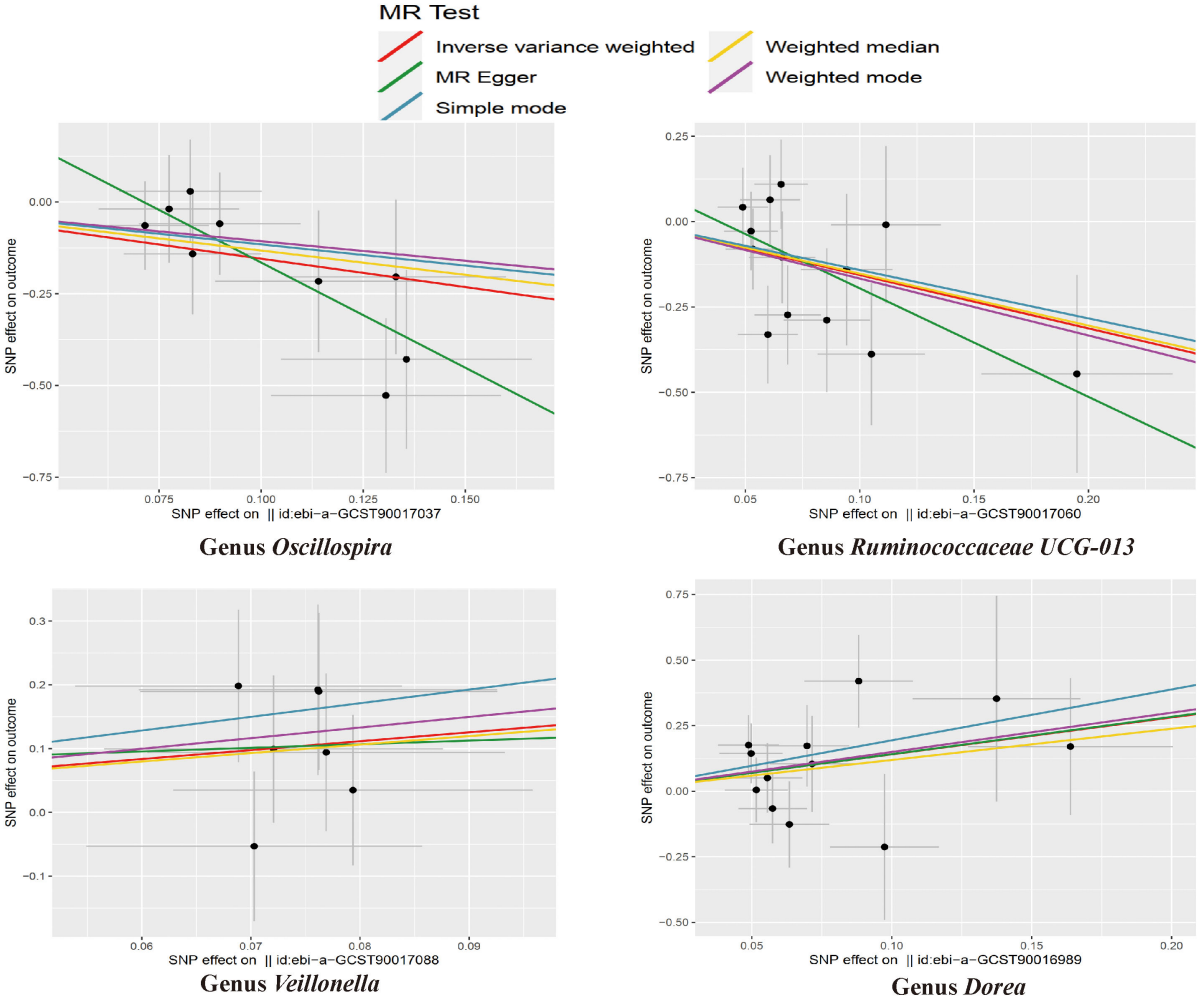
The scatter plots for the causal association between gut microbial taxa and NASH.

MR estimate for the association between gut microbiota and NASH was shown in **Table 2** and **Figure 7**. The scatter plots showed the causal relationship between gut microbiota and NASH in the five MR Methods (**Figure 8**). Sensitivity analyses, including Cochran’s Q test, MR-Egger, MR-PROSSO (shown in **Supplementary Table 5**), and Leave-one-out analyses (shown in **Figure 5**), did not provide any evidence of pleiotropy or heterogeneity (P > 0.05). Furthermore, inverse MR analyses did not reveal a causal effect of NASH on the aforementioned gut microbiota.

**Table 2:** MR estimate for the association between gut microbiota and NASH.

Bacterial taxa (Exposure)	MR method	SNP(N)	F-statistic	OR	95%CI	*P* value
*Oscillospira*	Inverse variance weighted	6	40.5	0.23	0.06-0.92	0.04
	MR Egger	6		0.00	0.00-0.69	0.11
	Weighted median	6		0.29	0.05-1.87	0.19
	Weighted mode	6		0.37	0.02-5.58	0.51
	Simple mode	6		0.33	0.02-5.09	0.46
*Ruminococcaceae UCG-013*	Inverse variance weighted	9	26.1	0.14	0.04-0.55	0.00
	MR Egger	9		0.05	0.00-1.52	0.13
	Weighted median	9		0.19	0.03-1.23	0.08
	Weighted mode	9		0.18	0.01-2.78	0.26
	Simple mode	9		0.25	0.01-5.00	0.39
*Veillonella*	Inverse variance weighted	4	45.5	5.79	1.08-31.10	0.04
	MR Egger	4		6.67E+05	0.00-1.15E+24	0.60
	Weighted median	4		12.22	1.56-95.53	0.02
	Weighted mode	4		12.79	0.52-316.73	0.22
	Simple mode	4		12.79	0.71-230.89	0.18
*Dorea*	Inverse variance weighted	9	24.8	5.57	1.20-25.76	0.03
	MR Egger	9		8.15	0.01-12410.89	0.59
	Weighted median	9		5.31	0.64-43.82	0.12
	Weighted mode	9		9.57	0.32-286.91	0.23
	Simple mode	9		8.32	0.26-269.84	0.27

MR, Mendelian randomization; SNP, Single nucleotide polymorphisms; N, Numbers; CI, Confidence interval.

## 4 Discussion

Through an MR analysis, we identified causal associations between ten gut microbial taxa (ranging from order to genus level) and NAFLD/NASH. Order Enterobacteriales, family *Enterobacteriaceae*, and genus *Lachnospiraceae UCG-004* and *Prevotella9* demonstrated strong causal relationships with NAFLD, with increased abundance corresponding to an increased risk of NAFLD. Moreover, *Dorea*, *Oscillospira*, *Ruminococcaceae UCG-013*, and *Veillonella* had significant causal relationships with NASH. Increased abundance of *Dorea* and *Veillonella* was associated with a heightened risk of NASH, whereas the people with higher abundance of *Oscillospira* and *Ruminococcaceae UCG-013* may have lower NASH risks. In addition, we also conducted a reverse MR study, suggesting that NAFLD may drive the increased abundance of *Holdemania* and *Ruminococcus2*.

*Prevotella* is a genus of Gram-negative bacteria that is part of the Bacteroidetes phylum and it is one of the most abundant microbiota in the human gut (Tett et al., 2021). Li et al. meta-analyzed six original studies and found that patients with NAFLD had an increased abundance of *Prevotella* compared to healthy people (SMD=1.89 [95% CI: 0.02, 3.76]) (Li et al., 2021). Based on previous research, our study accurately demonstrated that genus *Prevotella9* can lead to an increased risk of NAFLD. The family *Enterobacteriaceae*, part of the Enterobacteriales order and comprising common pathogens like E. coli and Salmonella (Janda and Abbott, 2021), has substantial implications in NAFLD due to its capacity to produce lipopolysaccharide (LPS) and endogenous ethanol to induce oxidative stress and inflammation in the liver while increasing intestinal permeability (DuPont and DuPont, 2011; Leclercq et al., 2014). Shen et al. found that family *Enterobacteriaceae* was enriched in the NAFLD group compared to healthy subjects and patients with significant fibrosis had a higher abundance of family *Enterobacteriaceae* (13.92% vs 2.07%; *P <*0.01) compared to those with F0/F1 fibrosis (Shen et al., 2017). The genus *Lachnospiraceae UCG-004*, belonging to the family *Lachnospiraceae*, has been found to be positively correlated with fasting blood glucose (FBG) and the Homeostatic Model Assessment for Insulin Resistance (HOME-IR) levels (Zhang et al., 2021), while blood glucose levels and insulin resistance have been shown to be significantly associated with NAFLD (Radu et al., 2023). Meanwhile, Adams et al. found that the abundance of family *Lachnospiraceae* was increased in patients with advanced fibrosis of NAFLD compared with controls and was associated with increased serum glycocholic acid and fecal deoxycholic acid concentrations (Adams et al., 2020).

NASH, a potentially severe stage of NAFLD that may progress to cirrhosis and hepatocellular carcinoma (Younossi et al., 2023), is tightly linked to dysbiosis of the gut microbiota (Pierantonelli and Svegliati-Baroni, 2019). However, the specific microbiota changes in NAFLD patients throughout different disease stages, including NASH, remain underexplored. Our study further elucidates the causal relationship between *Dorea*, *Oscillospira*, *Ruminococcaceae UCG-013*, and *Veillonella* and NASH through MR analysis.

Consistent with our study, Del Chierico et al. compared stool samples of children and adolescents with NAFL and NASH with healthy control group and found that proportion of *Oscillospira* decreased and proportion of *Dorea* increased in NASH group through multivariate analysis (Del Chierico et al., 2017). Interestingly, Del Chierico et al. also reported that *Ruminococcus* increased significantly in NAFLD and NASH patients compared with the control group, but Li et al. (2021) reported that *Ruminococcus* decreased in NAFLD patients after meta-analysis of several cross-sectional studies. At the same time, Shu et al. found that the abundance of genus *Ruminococcus2* increased significantly in NAFLD mice (Shu et al., 2023). Contradictory findings in various studies related to *Ruminococcus* abundance underscore the heterogeneity of relationships between different *Ruminococcaceae* family genera and NAFLD/NASH. Our two-way MR analysis identified the genus *Ruminococcaceae UCG-013* as negatively correlated with NASH and NAFLD as the risk factor for the augmentation of *Ruminococcus2*.

It is well known that SCFAs are important metabolites produced by *Oscillospira* through the fermentation of soluble dietary fiber in the intestinal tract. SCFAs nourish intestinal epithelial cells and help maintain the stability of intestinal barrier function (Rau et al., 2018). Additionally, SCFAs can improve hepatocyte steatosis by activating AMP-activated protein kinase, expressing fatty acid oxidation genes, and inhibiting macrophage-mediated inflammatory response (Skelly et al., 2019). The specific mechanism of *Dorea* in NASH progression has not been widely studied. However, *Dorea* is strongly associated with inflammatory bowel disease and is considered to have pro-inflammatory effects (Zhang et al., 2021). The genus *Veillonella* metabolizes lactic acid to propionic acid and is involved in bile acid metabolism (Loomba et al., 2021). Mohammadi et al. compared the gut microbial composition of patients with presumed NASH to patients with NAFLD and found that the genus *Veillonella* was significantly more abundant in patients presumed to have NASH (Mohammadi et al., 2022). Additionally, some studies have found that *Veillonella* is significantly enriched in the gut microbiota of patients with liver cirrhosis, further indicating that *Veillonella* may be a key genus in the development of NASH (Chen et al., 2016; Oh et al., 2020).

In previous studies, Zhang et al. and Li et al. also found a causal relationship between NAFLD and gut microbiota by MR analysis. Nevertheless, the dataset we selected was different from theirs and we further explored the association between gut microbiota and NASH, reflecting the potential role of different microbial taxa in promoting the progression of mild NAFLD to NASH (Li et al., 2023; Zhang et al., 2023). Finally, in the inverse MR analysis, NAFLD appeared to affect the abundance of microbial taxa, which was consistent with the bidirectional effect of the gut-liver axis, which had not been reported in previous studies.

It must be acknowledged that there are some deficiencies in our research. (i) Since the number of IVs fulfilling the strict threshold (*P <*5×10^−8^) was extremely small, a relatively lenient threshold (*P <*1×10^−5^) was adopted for screening IVs. (ii) This study included individuals of essentially European ancestry, so extrapolating the findings to other populations is limited. (iii) Summary statistics for gut microbial taxa was profiled by targeting three distinct variable regions of the 16S rRNA gene. Bacterial taxa were only analyzed at the genus level but not at a more specialized level such as species or strain levels. When microbiota genome-wide association studies use more advanced shotgun metagenomic sequencing analysis, the results can be more specific and accurate. (iv) The GM-related GWAS summary-level dataset included in this study was based on 16S rRNA sequencing. Therefore, further analysis based on large-scale studies with more advanced methods, such as metagenomics sequencing, is required in the future to evaluate at the species-level. (v) Since the MR analysis is based on an untestable assumption, further experimental and clinical validation studies are crucial to test the clinical significance of microbial species.

In summary, we performed a bidirectional two-sample MR analysis using published GWAS summary data to identify 6 types of microbial taxa that contribute to the development of NAFLD/NASH and also identified 2 types of microbial taxa that causally decrease NASH risk. In addition, we determined that NAFLD can affect the abundance of gut microbiota reversely. Future studies are warranted to further dissect the potential mechanisms of action between specific microbial taxa and NAFLD and NASH.

## Data availability statement

Publicly available datasets were analyzed in this study. This data can be found here: https://www.finngen.fi/en/access_results and https://mibiogen.gcc.rug.nl.

## Ethics statement

Ethical approval was not required for the studies involving humans because the original dataset used is fully public and allowed to be used, and all have been ethically approved. The studies were conducted in accordance with the local legislation and institutional requirements. Written informed consent for participation was not required from the participants or the participants’ legal guardians/next of kin in accordance with the national legislation and institutional requirements because the original dataset used is fully public and allowed to be used, and all have informed consent.

## Author contributions

HW: Conceptualization, Data curation, Investigation, Visualization, Writing – original draft. QZ: Data curation, Methodology, Software, Visualization, Writing – original draft. SZ: Data curation, Investigation, Validation, Writing – review & editing. TZ: Data curation, Writing – original draft. CD: Methodology, Writing – original draft. JY: Investigation, Writing – original draft. JP: Investigation, Writing – original draft. CC: Investigation, Project administration, Writing – review & editing. JL: Funding acquisition, Project administration, Validation, Writing – review & editing.
