# Supplementary material for: Association between gut microbiota and NAFLD/NASH: a bidirectional two-sample Mendelian randomization study
Source: Front Cell Infect Microbiol. 2023 Dec 1;13:1294826. doi: 10.3389/fcimb.2023.1294826 (PMC10722258; doi:10.3389/fcimb.2023.1294826)
Supplement: Supplementary file 1 [file DataSheet_1.docx]

**Supplementary table 1. The baseline information for NAFLD, NASH, and microbiome cohorts**

|  | **NAFLD cohort (n=377277)** | **NASH cohort (n=377277)** | **Microbiota cohort (n=18320)** |
| --- | --- | --- | --- |
| Number of case | 2275 | 157 | NA |
| Male | 1000 | 62 | 8103 |
| Female | 1275 | 95 | 10217 |
| Mean age (years) | 53.3 | 57.1 | 47.5 |
| Mean BMI (kg/m^2^) | NA | NA | 25.2 |

**Supplementary table 2. The sensitivity analyses of causality between GM taxa and NAFLD based on MR results**

| **Bacterial taxa** | **Q-P value** | **Egger interpret** | **Egger intercept - P value** | **MR-PRESSO test** |
| --- | --- | --- | --- | --- |
| family *Enterobacteriaceae* | 0.53 | -0.02 | 0.68 | 0.54 |
| order Enterobacteriales | 0.53 | -0.02 | 0.68 | 0.54 |
| genus *Lachnospiraceae* | 0.44 | -0.04 | 0.31 | 0.47 |
| genus *Prevotella 9* | 0.54 | 0.00 | 0.95 | 0.56 |

**MR-PRESSO test, the mendelian randomization pleiotropy residual sum and outlier test; Q-P value, Cochran's Q test P-value.**

**Supplementary table 3. MR estimate for the association between NAFLD and gut microbiota**

| **Bacterial taxa (Outcome)** | **MR method** | **SNP(N)** | **F-statistic** | **OR** | **95%CI** | **P value** |
| --- | --- | --- | --- | --- | --- | --- |
| Genus *Holdemania* | Inverse variance weighted | 13 | 24.8 | 1.05 | (1.01, 1.09) | 0.01 |
|  | MR Egger | 13 |  | 1.14 | (0.90, 1.45) | 0.30 |
|  | Weighted median | 13 |  | 1.04 | (0.96, 1.12) | 0.34 |
| Genus *Ruminococcus2* | Inverse variance weighted | 12 | 24.5 | 1.08 | (1.03, 1.13) | 0.00 |
|  | MR Egger | 12 |  | 0.98 | (0.81, 1.18) | 0.81 |
|  | Weighted median | 12 |  | 1.08 | (1.02, 1.16) | 0.01 |

**MR, Mendelian randomization; SNP, single nucleotide polymorphisms; N, numbers; CI, Confidence interval.**

**Supplementary table 4. The sensitivity analyses of causality between NAFLD and GM taxa based on MR results**

| **Bacterial taxa** | **Q-P value** | **MR Egger-interpret** | **Egger intercept - P value** | **MR-PRESSO test** |
| --- | --- | --- | --- | --- |
| Genus *Holdemania* | 0.97 | -0.02 | 0.49 | 0.70 |
| Genus *Ruminococcus2* | 0.45 | 0.01 | 0.64 | 0.20 |

**MR, Mendelian randomization; MR-PRESSO test, the mendelian randomization pleiotropy residual sum and outlier test; Q-P value, Cochran's Q test P-value.**

**Supplementary table 5. The sensitivity analyses of causality between GM taxa and NASH based on MR results**

| **Bacterial taxa** | **Q-P value** | **MR Egger-interpret** | **Egger intercept - P value** | **MR-PRESSO test** |
| --- | --- | --- | --- | --- |
| Genus *Oscillospira* | 0.71 | 0.41 | 0.13 | 0.61 |
| Genus *Ruminococcaceae* | 0.46 | 0.12 | 0.33 | 0.50 |
| Genus *Veillonella* | 0.72 | 0.06 | 0.95 | 0.74 |
| Genus *Dorea* | 0.58 | 0.00 | 0.99 | 0.61 |

**MR, Mendelian randomization; MR-PRESSO test, the mendelian randomization pleiotropy residual sum and outlier test; Q-P value, Cochran's Q test P-value.**
